# Child Negative Emotionality and Parental Harsh Discipline in Chinese Preschoolers: The Different Mediating Roles of Maternal and Paternal Anxiety

**DOI:** 10.3389/fpsyg.2017.00339

**Published:** 2017-03-07

**Authors:** Xiaopei Xing, Hongli Zhang, Shuhui Shao, Meifang Wang

**Affiliations:** ^1^Beijing Key Laboratory of Learning and Cognition, Department of Psychology, Research Center for Child Development, Capital Normal UniversityBeijing, China; ^2^College of Elementary Education, Capital Normal UniversityBeijing, China

**Keywords:** child negative emotionality, parental anxiety, psychological aggression, corporal punishment, China

## Abstract

Previous research has suggested that harsh discipline is still prevalent in modern Chinese families and it is necessary to explore the cause and the potential mechanisms of Chinese parental use of harsh discipline. This study examined the mediating effects of parental anxiety in the relations between child negative emotionality and parental harsh discipline in China. Using a sample of 328 Chinese father-mother dyads with their young children, findings revealed that maternal anxiety mediated the relations between child negative emotionality and maternal psychological aggression and corporal punishment, but the mediating effects of paternal anxiety on the relations between child negative emotionality and paternal harsh discipline was not significant. The findings provide an important supplement and extension to previous examinations of the factors associated with Chinese parental use of harsh discipline and its mechanisms.

## Introduction

Despite numerous studies have demonstrated that children exposed to parental harsh discipline (e.g., psychological aggression, corporal punishment, and physical abuse) are at risk for a number of negative developmental outcomes, including aggression, anxiety, and depression (i.e., Fine et al., 2004; Xing et al., 2011; Xing and Wang, 2013), harsh discipline is still the prevalent parental disciplinary technique in modern Chinese families. According to a large sample survey conducted in Mainland China (Wang and Liu, 2014), more than 80% of parents with children aged 3–6 reported the use of psychological aggression, and about 70% of parents reported the use of corporal punishment during the previous year. Furthermore, it was also found that the frequency of parental use of psychological aggression toward the preschool-aged children was about 14 times per year, and the mean frequency of corporal punishment was about 7 times per year. The high prevalence and frequency of harsh discipline in Mainland China and the growing evidence on the negative consequences of such parenting practices necessitate research to determine the causes of harsh discipline. To date, some research has shown that parental use of harsh discipline is likely heavily dependent on child heritable characteristics, with temperament key among them (Gershoff, 2002). Child temperament traits may influence the types of discipline they receive, a phenomenon referred to as an *evocative gene–environment correlation* (Plomin et al., 1977; Scarr and McCartney, 1983; Scarr, 1992; Reiss, 1995).

In temperament research, the notion that child negative emotionality may have an evocative effect upon parenting behavior has become a central issue as negative emotionality is considered to be the core dimension of the difficult temperament concept (Lee and Bates, 1985; Bates, 1989; Prior, 1992; Shiner, 1998). *Negative emotionality* refers to irritability, negative mood, (un)soothability, and high-intensity negative reactions. Given that negative emotionality is typically viewed adversely by adult (Ho, 1986; Bates, 1989), irritable, demanding, or withdrawn children are more likely to elicit negative, angry or coercive, and highly controlling parenting. For example, Bates et al. (1995) and Kochanska et al. (2004) found that children's temperament difficultness and negative emotionality were linked to unresponsive, harsh, and controlling parenting (Bates et al., 1995; Kochanska et al., 2004). Day et al. (1998) found that children described by their parents as having fussy or irritable temperaments tend to be spanked more than children reported to have happy or cheerful temperaments (Day et al., 1998). In a meta-analysis of 62 Western studies, Paulussen-Hoogeboom et al. (2007) found that although the relation between child negative emotionality and parental restrictive control, a type of control characterized by high power assertion, negativity, intrusiveness, hostility, or overcontrolling behavior, was weak, this relation was significant for infants and preschoolers, but not for 1-year-olds, and for samples with predominantly first-born children. Furthermore, they found that the relation depended on the measuring methods, and only studies using parent report or composite measures (including observation and parent report) to assess both negative emotionality and restrictive parenting yielded significant effect sizes (Paulussen-Hoogeboom et al., 2007). Despite such progress, almost all of the studies in this area have been conducted in Western cultures; there is a dearth of research on the effects of child negative emotion on parental harsh discipline in Chinese society. To date, only one cross-cultural study examined the linkages between child temperament and parenting styles with samples from Beijing, China, and the western United States and found that child negative emotionality was positively associated with authoritarian parenting in both cultures (Porter et al., 2005). Nevertheless, some questions have been raised regarding the cross-cultural generalizability of the antecedents and the mechanisms of harsh parenting. For example, harsh discipline is more likely to be used by parents in Asian cultures and some ethnic minority (e.g., Hispanic or African American; Brooks-Gunn and Markman, 2005), and these families often value conformity in their children more than Caucasian White families do (Ispa et al., 2004). Possibly, parents from Asian cultures or ethnic minorities would exercise greater controlling strategies when confronted with a fussy or irritable child because they do not want to encourage their children's undesirable behaviors and because they are less inclined to react from their child's point of view. Moreover, given that authoritarian parenting comprises multiple parenting practices, including but not limited to parental psychological aggression and corporal punishment, it remains unclear if child negative emotionality would elicit parental use of psychological aggression and corporal punishment in Chinese families.

Another important next step in understanding the link between child negative emotionality and parental harsh discipline is to identify the potential mechanisms underlying this association. One important way in which child negative emotionality may affect parental discipline is through increased parental negative emotion, such as anxiety. Although there is no direct support for the effects of children's negative emotionality on parental anxiety, children's difficult temperament has been shown to be associated with mothers' low level of psychological well-being (Solmeyer and Feinberg, 2011), such as mothers' elevated stress levels (McBride et al., 2002), more psychosocial problems (Sheeber and Johnson, 1992), higher levels of depression (Cutrona and Troutman, 1986), and low self- efficacy (Teti and Gelfand, 1991; Porter and Hsu, 2003). Because of the confluence of theoretical perspectives emphasizing the centrality of child negative emotionality to parental mood disorders, the present study chose to focus on negative emotionality to test its effects on parental anxiety. It was expected that parents would experience high levels of anxiety when they must cope with children who are chronically fussy, irritable, and difficult to read.

Consistent with this conceptualization, it has been suggested that negative emotional feelings such as anxiety within parents, if not well-regulated, can further influence parents' cognitions, motivations, and parenting behavior and may become a major feature of chronically harsh parenting (Dix, 1991; Scaramella and Leve, 2004; Chen et al., 2015). Indeed, Ginsburg and Schlossberg (2002) suggested that high levels of anxiety in parents could interfere with the development of parents' adaptive coping skills, which might lead to specific “anxiety-enhancing” parenting behaviors, such as rejection and (over) control. Previous studies into negative or abusive parenting also lend some support to the associations between parental anxiety and harsh discipline. Bögels and Brechman-Toussaint (2006) showed that parental anxiety could drive over-controlling, negative parenting behaviors. Abusive parents compared to non-abusive control group were found to report higher levels of anxiety (Whipple and Webster-Stratton, 1991). Based on the above analyses, we proposed that associations between child negative emotion and parental harsh discipline might be indirect through (or mediated by) parental anxiety. To our knowledge, only one study to date examined the mediating role of maternal well-being (including depressive symptoms and self-esteem) in the associations between child difficult temperament and maternal parenting style, and their results indicated that child negative emotionality was only directly associated with maternal psychological and behavioral control but not indirectly via parental well-being (Laukkanen et al., 2014).

Pertinent social contexts within which the Chinese sample was taken need to be delineated to better understand the relations between child negative emotionality and parental harsh discipline in China. Traditionally, some characteristics of the Chinese culture stemming from Confucian principles, such as the restraint of emotion, parental authority, and parental training of children, may facilitate parental use of harsh discipline when they interacted with highly negative child (Chao, 1994). Specifically, individuals are encouraged to govern negative emotion expression during interpersonal interactions in Chinese societies, and the expression of emotions, particularly negative emotions, is often regarded as shameful to self and family (Wang et al., 2006). Moreover, given that Chinese parents generally have high expectations of their children and want them to bring honor to their family name, they may be less tolerant of children who are temperamentally high in negative emotionality. Furthermore, as the Chinese proverb says, “Beating and scolding is the emblem of love,” traditional Chinese culture views parental psychological and physical aggression as indications of parental love and concern (Chao, 1994; Simons et al., 2000) and these disciplinary techniques are generally accepted by Chinese parents. Thus, it may be reasonable to speculate that Chinese parents would be more anxious and more likely to implement psychological aggression or corporal punishment to suppress child's negative emotions when child exhibited distress, anger, or fear.

Moreover, Belsky and Barrends (2002) called for research that includes both mothers and fathers. However, most empirical work on the effects of child characteristics on parental well-being or parental discipline has focused on mothers, less attention has been paid to fathers. Nevertheless, the available evidence from studies investigating the relations between child temperament and parental well-being showed some mother-father differences. For example, one community-based study has found that low positive emotionality in children is associated with maternal, but not with paternal, mood disorder (Durbin et al., 2005). With regard to parental harsh discipline, findings from Chen et al. (2007), based on Chinese elementary school-aged children sample, indicated that child negative emotion was significantly linked to maternal but not paternal high levels of rejection, denial, or punishment. A limitation of previous research is that few studies consider both mothers and fathers in the same statistical models, making it difficult to explicitly test whether the relation patterns between child negative emotionality and parental harsh discipline and the potential mechanisms underlying these relations are different for fathers and mothers.

In traditional Chinese culture, the appropriate roles of fathers and mothers in childrearing have been refined. For example, the Chinese proverb “strict father, kind mother” implies greater control exerted by fathers and greater emotional support or warmth manifested by mothers. Chinese fathers often assume more disciplinary duties in child socialization than do mothers, “It is the father's fault if a child is not adequately educated,” as stated in the famous “Three Character Classic” (Mo, 1996; Chen et al., 2002). Within such traditional cultural context, fathers may be blamed and may feel a sense of responsibility or even guilt when something goes wrong with their children. Thus, it is conceivable that compared to mothers, Chinese fathers are more likely to be anxious and to use harsh discipline toward a difficult child. However, while past studies (Kwok and Wong, 2000; Tam and Lam, 2003) confirmed the different roles played by mothers and fathers in Chinese families, it is undeniable that in most families, mothers are the primary caregivers and they spend more time and effort in caring their children. A recent study by Ma et al. (2013) indicated that modernization and westernization have transformed the parental roles/functions of Chinese parents from “a stern father and a warm and caring mother” to “an under-involved father and an over-involved mother,” with the latter assuming both the caring and disciplinary roles in child rearing. More involvement and dual parenting roles may lead mothers to have more chance to be influenced by the child. In this regard, it also can be expected that child negative emotionality would be more strongly associated with maternal but not paternal anxiety and harsh discipline. Thus, in order to make clear the potential father-mother differences in these relations and mechanisms, the current study would examine the mediating role of both paternal and maternal anxiety in the relations between child negative emotionality and paternal and maternal harsh discipline in the same model.

In sum, the current study collected data from a large sample of Chinese mothers and fathers with preschool children and examined the potential mediating role of parental anxiety in the associations between child negative emotionality and parental harsh discipline (psychological aggression and corporal punishment) and parent gender differences in the mediating mechanisms. We expected that child negative emotionality would be positively associated with both paternal and maternal psychological aggression and corporal punishment, and paternal anxiety or maternal anxiety would mediate these associations. We also expected some father-mother differences in these associations and mechanisms. We believed that to some extent this study would help understand the child-effects on parental harsh discipline in the Chinese context.

## Methods

### Participants

The sample consisted of 328 Chinese preschool-aged children and their parents (49.4% boys, mean age = 4.11 years, *SD* = 0.33). The mean ages of the fathers and mothers were 36.41 (*SD* = 4.34; age range = 23–51 years) and 34.16 (*SD* = 3.60; age range = 26–51 years), respectively. Nearly 84% of the children were reported to have no siblings. Mother's median income ranged from ¥6,000 to ¥10,000 RMB per month and Father's median income ranged from ¥10,000 to ¥15,000 RMB per month. In terms of education, about 10% of the fathers and the mothers completed high school or less, 62.7% of the fathers and 65% of the mothers had a college/university education, and 27.8% of the fathers and 24.9% of the mothers had completed at least some postgraduate education. Concerning employment, 22.0% of the fathers and 21.7% of the mothers were employed in working-class jobs (e.g., factory workers), whereas 39.7% of the fathers and 36.4% of the mothers held a professional, managerial, or technical position.

### Procedure

The children and their parents were recruited from four public kindergartens in Beijing, the capital city of China. Parental consent was obtained for all participating families; Parents were also made aware of the voluntary and confidential nature of this research before administration of the study measures. After obtaining parental informed consent, two packets (one for the mother and one for the father) containing the parental questionnaires were sent home with the participating children. The parents were asked to fill the questionnaires out independently at home and handed the completed form to their child's teacher. The data collection procedures for this study were approved by the Ethics Committee of Educational College of Capital Normal University.

### Measures

#### Parental harsh discipline

Parental harsh discipline was assessed via a Chinese version of the Parent–Child Conflict Tactics Scales (CTSPC; Straus et al., 1998; Xing and Wang, 2013). It was often used to assess mother-to-child and father-to-child discipline strategies, which includes five subscales: Non-violent discipline (4 items), psychological aggression (5 items), corporal punishment (6 items), physical maltreatment (3 items), and extreme physical maltreatment (4 items). In this study we focused on the psychological aggression and corporal punishment subscales given their high prevalence in Chinese families (Wang and Liu, 2014). Mothers and fathers were asked to report separately about how many times they implemented specific behaviors with their children in the previous year. The response options included never (0); once (1); twice (2); 3–5 times (4); 6–10 times (8); 11–20 times (15); more than 20 times (25). Mothers and fathers' scores in psychological aggression and corporal punishment subscales were calculated by summing the frequency scores of the subscale items separately. In the present study, the Cronbach's alpha coefficients for the psychological aggression subscale were 0.62 for mother-report and 0.61 for father-reports, respectively. The Cronbach's alpha coefficients for the corporal punishment subscale were 0.62 for father-report and 0.68 for mother-report. These alphas are consistent with those obtained in previous research (Straus et al., 1998; Fine et al., 2004; Wang et al., 2014).

#### Parental anxiety

The Chinese version of the DASS-21 was chosen to assess paternal and maternal anxiety in the current study (Wang et al., 2011). It was a 21-item self-report questionnaire including three subscales: Depression, anxiety, and stress. Each subscale had seven items and the anxiety subscale was selected in this study. Fathers and mothers were separately asked to rate the frequency/severity of each item that they have experienced over the past week and circle their response from four short options ranging from 0 (Did not apply to me at all) to 3 (Applied to me very much or most of the time). The scores for anxiety were calculated by summing the scores of seven items in this subscale, separately for mothers and fathers. The Cronbach's alpha coefficients for anxiety subscale were 0.63 for mother report and 0.70 for father report, in the present study.

#### Child negative emotionality

The Chinese version of Child Behavior Questionnaire–Very Short Form (CBQ-VSF) was used to assess children's negative emotionality, which can be used to assess 3–8 year-olds' temperament (Liang et al., 2009). It comprises 36 items assessing the following three scales: Effortful control, surgency, and negative emotionality. The negative emotionality subscale was used in current study. Mothers were selected to rate their children's negative emotionality in the past 6 months. The response options were from 1 (extremely untrue of your child) to 7(extremely true of your child), plus a not applicable response option if the description of their child's reactions did not occur within the time. In the present study, the Cronbach's alpha coefficient for the negative emotionality subscale was 0.62.

#### Demographic characteristics

Demographic information was collected via mother report. This study included items related to the child's age and gender, paternal/maternal education, and current paternal/maternal income. Parental education was rated on a 7-point scale ranging from 1 (less than 3 years) to 7 (master's or doctoral degree) and month income was rated on a 7-point scale ranging from 1 (less than ¥1,500) to 7 (more than ¥20,000). For each family, the responses on education and current income of both mother and father were standardized and averaged, yielding a global index of family socioeconomic status, with higher scores indicating higher family socioeconomic status.

## Results

### Preliminary analyses

Analyses of demographic differences on all study variables were conducted for child gender, child age, and family socioeconomic status. As shown in Table 1, boys were more likely than girls to experience paternal corporal punishment, *t*_(326)_ = 2.10, *p* < 0.05. In addition, the results showed that maternal psychological aggression, *r* = −0.11, *p* < 0.05, and corporal punishment, *r* = −0.17, *p* < 0.01, were negatively associated with family SES. No other statistically significant differences were found on variables of interest, all *p* > 0.05.

**Table 1:** **Descriptive statistics for child negative emotionality, parental anxiety, and parental harsh discipline for boys and girls**.

**Variables**	**Boys**		**Girls**		***t***	**Cohen's d**
	**M**	***SD***	**M**	***SD***	**(Gender)**	
Child negative emotionality	3.74	0.75	3.81	0.72	−0.81	−0.10
Maternal anxiety	1.70	1.77	1.56	2.04	0.65	0.07
Paternal anxiety	1.41	1.99	1.58	2.21	−0.71	−0.08
Maternal psychological aggression	19.10	16.18	18.45	16.85	0.36	0.04
Paternal psychological aggression	15.72	15.47	12.72	12.63	1.93^+^	0.21
Maternal corporal punishment	9.32	11.99	8.31	12.86	0.74	0.08
Paternal corporal punishment	7.25	9.37	5.06	9.49	2.10^*^	0.23

*t-values are for comparisons of boys and girls; Cohen's d is the effect size for group differences*.

+ *p < 0.1*,

* *p < 0.05*.

Table 2 presents the bivariate correlations among all study variables for full samples. As shown, children's negative emotionality was positively correlated with both parental anxiety and maternal psychological aggression and corporal punishment (but not with paternal psychological aggression and corporal punishment). Maternal anxiety was positively correlated with maternal psychological aggression and corporal punishment. Paternal anxiety was positively correlated with paternal psychological aggression (but not with paternal corporal punishment).

**Table 2:** **Correlations among child negative emotionality, parental anxiety, and parental harsh discipline**.

	**1**	**2**	**3**	**4**	**5**	**6**	**7**
1. Child negative emotionality	–						
2. Maternal anxiety	0.15^**^	–					
3. Paternal anxiety	0.12^*^	0.26^**^	–				
4. Maternal psychological aggression	0.16^**^	0.33^**^	0.05	–			
5. Paternal psychological aggression	0.08	0.11^*^	0.15^**^	0.34^**^	–		
6. Maternal corporal punishment	0.11^*^	0.22^**^	0.05	0.62^**^	0.25^**^	–	
7. Paternal corporal punishment	0.10	0.05	0.04	0.26^**^	0.49^**^	0.32^**^	–

* *p < 0.05*,

** *p < 0.01*.

### Parental anxiety as mediating variables

Two structural equation models (one for parental psychological aggression and one for parental corporal punishment) were conducted in Mplus 7.0 to examine the mediating effects of parental anxiety on the association between child negative emotionality and parental harsh discipline after controlling family SES. Full maximum likelihood estimation was used to handle missing data. Bias-corrected bootstrap method was performed to evaluate the significance of the mediated paths in the current study. The model included two direct paths from child negative emotionality to maternal and paternal harsh discipline variables, and indirect paths via parental anxiety. Two models were supported by adequate goodness-of-fit statistics, for parental psychological aggression model, χ^2^*/df* = 0.95, *RMSEA* = 0.00, *TLI* = 1.01, *CFI* = 1.00, *SRMR* = 0.03; for parental corporal punishment model, χ^2^*/df* = 1.01, *RMSEA* = 0.01, *TLI* = 1.00, *CFI* = 1.00, *SRMR* = 0.03.

**Figure 1:**
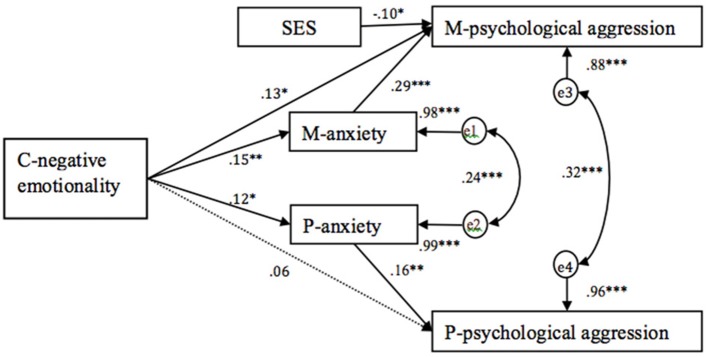
**Mediation model testing the direct impacts of children's negative emotionality on parental psychological aggression, and indirect impacts of children's negative emotionality on parental psychological aggression via parental anxiety after controlling family SES (standardized estimates)**. C- negative emotionality, children's negative emotionality; M-anxiety, maternal anxiety; P-anxiety, paternal anxiety; M-psychological aggression, maternal psychological aggression; P-psychological aggression, paternal psychological aggression. ^*^p < 0.05, ^**^p < 0.01, ^***^p < 0.001.

As shown in Figure 1, the direct path from child negative emotionality to maternal psychological aggression, β = 0.13, *p* < 0.05, but not paternal psychological aggression, β = 0.06, *p* > 0.05, was significant. Child negative emotionality was significantly associated with parental anxiety for both mothers and fathers, mothers: β = 0.15, *p* < 0.01; fathers: β = 0.12, *p* < 0.05. Parental anxiety was significantly associated with their psychological aggression for both mothers, β = 0.29, *p* < 0.001, and fathers, β = 0.16, *p* < 0.01. A bias-corrected bootstrap 95% confidence interval (CI) indicated that the indirect effect of child negative emotionality on maternal psychological aggression via maternal anxiety was significant, *standardized indirect estimate* = 0.04, *p* < 0.05, 95% CI: 0.02–0.07, but the indirect impact of child negative emotionality on paternal psychological aggression via paternal anxiety was not significant, *standardized indirect estimate* = 0.02, *p* > 0.05, 95% CI: 0.00–0.04.

**Figure 2:**
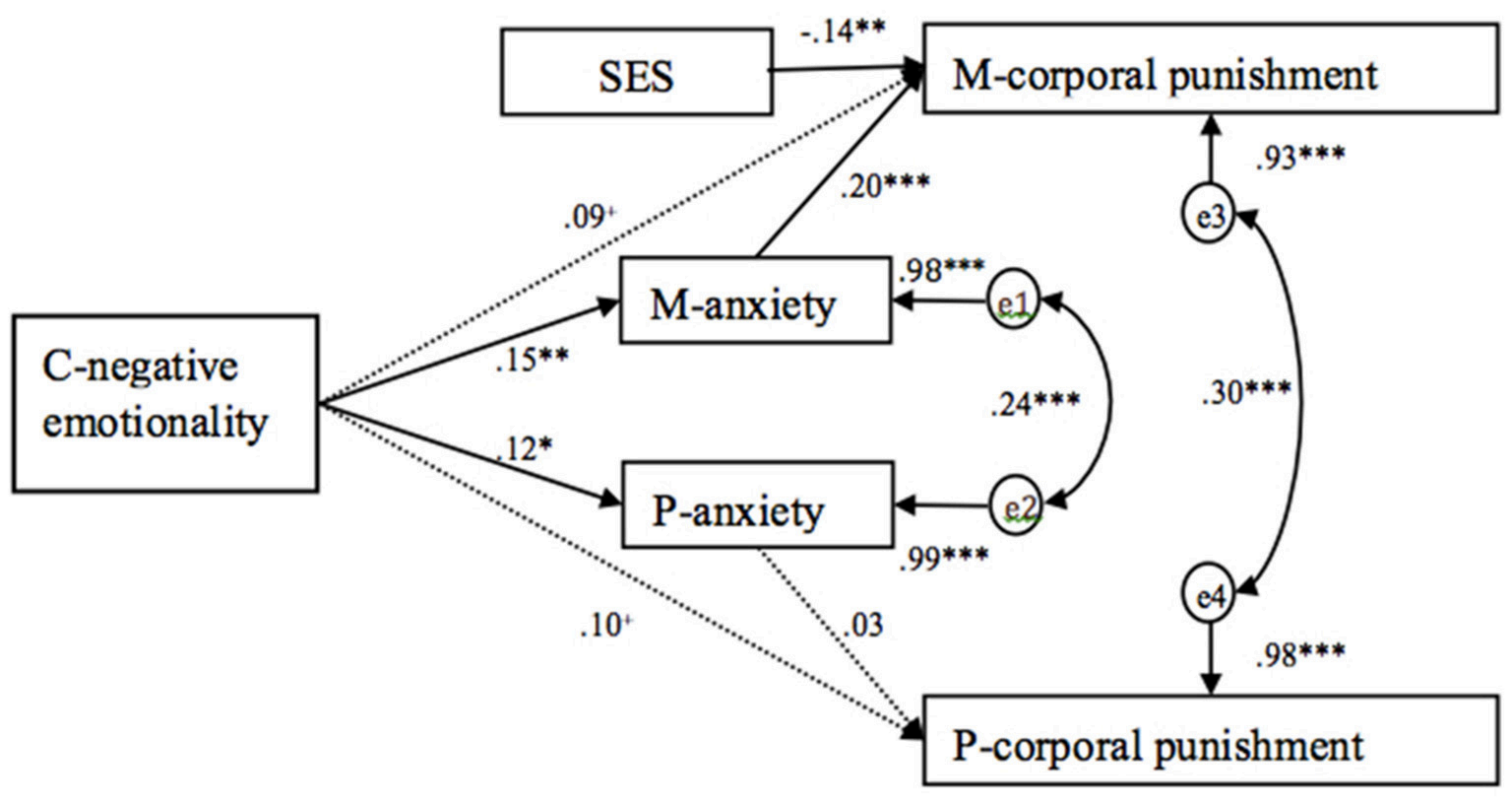
**Mediation model testing the direct impacts of children's negative emotionality on parental corporal punishment, and indirect impacts of children's negative emotionality on parental corporal punishment via parental anxiety after controlling family SES (standardized estimates)**. C- negative emotionality, children's negative emotionality; M-anxiety, maternal anxiety; P-anxiety, paternal anxiety; M-corporal punishment, maternal corporal punishment; P-corporal punishment, paternal corporal punishment. ^+^*p* < 0.1, ^*^p < 0.05, ^**^p < 0.01, ^***^p < 0.001.

As shown in Figure 2, child negative emotionality was not significantly directly associated with maternal and paternal corporal punishment, mothers: β = 0.09, *p* > 0.05; fathers: β = 0.10, *p* > 0.05, but was significantly associated with maternal and paternal anxiety, mothers: β = 0.15, *p* < 0.01; fathers: β = 0.12, *p* < 0.05. Parental anxiety was significantly associated with maternal but not paternal corporal punishment, mothers: β = 0.20, *p* < 0.001, fathers: β = 0.03, *p* > 0.05. A bias-corrected bootstrap 95% confidence interval (CI) indicated that the indirect effect of child negative emotionality on maternal corporal punishment via maternal anxiety was significant, *standardized indirect estimate* = 0.03, *p* < 0.05; 95% CI: 0.01–0.05, but the effect of child negative emotionality on paternal corporal punishment via paternal anxiety was not significant, *standardized indirect estimate* = 0.00, *p* > 0.05, 95% CI: −0.01–0.01.

## Discussion

The current study examined the mediating role of parental anxiety in the associations between child negative emotionality and parental harsh discipline in Chinese societies and explored the potential father-mother differences in the mediating mechanisms. The findings demonstrated that child negative emotionality was significantly associated with maternal but not paternal psychological aggression and corporal punishment. Furthermore, it was found that maternal anxiety mediated the associations between child negative emotionality and maternal psychological aggression and corporal punishment, but the indirect effects of child negative emotionality on the paternal harsh discipline via paternal anxiety were not significant. We now turn to a more detailed discussion of the major findings.

According to Scarr and McCartney (1983), children's unique characteristics provide an important context for parenting. Previous empirical studies have shown that children's difficultness or negative affect, particularly irritability, tends to elicit more harsh or inconsistent disciplinary responses from parents (Yang et al., 2004; Ganiban et al., 2011). Consistent with these findings, although the present data were correlational, our results to some extent found that child characteristics such as negative emotionality played a significant role in explaining why some children are more likely than others to be harshly disciplined. Moreover, expanding on the previous research by including mothers and fathers and by examining psychological aggression and corporal punishment separately, the current study found that child negative emotionality was significantly associated with maternal but not paternal harsh discipline, and this pattern held for both psychological aggression and corporal punishment. This father-mother difference may be explained by the fact that, on the one hand, mothers are typically the children's primary caregivers in Chinese families and they spend more time than do fathers in interaction with their children (Chang et al., 2003). In Chinese societies in which the cultural norms includes spanking or scolding children “when necessary,” it is thus understandable that due to more time spent in caregiving, mothers compared to fathers would more often be in the role of disciplinarian and may be more likely to be influenced by the child characteristics, such as children's display of negative emotion, as a result, being more likely to perform harsh disciplinary acts; On the other hand, young children tend to be closer to their mothers but often perceive their fathers as relatively distant authority figures, which may lead children to express more negative emotion toward their emotional-close mothers rather than toward their emotional-distant fathers. As a result, mothers may be more likely to engage in psychological or physical aggressive discipline in response to high levels of negative emotionality in children.

To further clarify the process of the influence of child negative emotionality on parental use of psychological aggression or corporal punishment, we conducted two structural equation models to test whether parental anxiety would mediate above associations. The results indicated that child negative emotionality was indirectly associated with maternal harsh discipline via maternal anxiety. It is possible that due to maternal more involvement and their caring and disciplinary roles in childrearing, children's intense emotions, irritability, and tendency to be difficult to soothe would be more likely to tax on maternal psychological or emotional resources and thus would place mothers in anxious and distressed emotional states. Meanwhile, within the Chinese cultural context emphasizing the restraint of emotion and less accepting children's negative emotions (Chen and Uttal, 1988; Lin and Fu, 1990; Chao, 1994; Chuang and Su, 2009), it is imperative for mothers to take immediate action to suppress the child's negative emotion. The immediacy of the parenting action as well as the anxious feelings may increase the likelihood that mothers would engage in rapid and automatic information processing, which in turn may induce the occurrence of reactive, easily instituted, and parent-centered tactics such as psychological aggression and corporal punishment rather than planful, reasoned out, and child-centered disciplinary tactics (e.g., inductive reasoning) that commonly require greater patience to implement (Grusec and Goodnow, 1994; Holden et al., 1998).

In addition, it is also important to note that although child negative emotionality was significantly associated with paternal anxiety, the mediating effects of paternal anxiety in the associations between child negative emotionality and paternal harsh discipline was not supported. This finding suggested that paternal psychological aggression and corporal punishment may not occur in response to child evocative characteristics or may not be influenced by their own emotional state. According to previous research, paternal use of corporal punishment may be associated with other factors in parents' distal as well as proximal contexts (e.g., childhood discipline; see Wang et al., 2014). Unfortunately, the constructs assessed in our study limited us to explore this issue. Future research may help to identify intervention targets that could diminish paternal use of harsh discipline.

Several limitations of this study should be noted. First, with correlational data, it is very difficult to identify causal relations. Although in the present study we made the choice to examine the potential child influence on parental anxiety and disciplinary behavior, it is possible that the direction of the effects may be reverse, for example, parents who are apt to use harsh discipline or who are more anxious may be more likely to elicit negative emotions from their children (Wang et al., 2016). Moreover, bidirectional relations should also be considered, for example, children high in negative emotionality may be more vulnerable to the adverse effects of harsh discipline, while in turn, harsh discipline would also predict increases in children's negative emotionality (Kiff et al., 2011; Wang and Liu, 2017). Thus, longitudinal research that collects data at multiple time points and controls for the stability or change in each variable may provide the promising evidence for the relations among child temperament, parental anxiety and harsh discipline. A second limitation concerns the use of maternal reports to measure child negative emotionality, maternal harsh discipline and maternal anxiety. Because the primary caregiver reported on both the child's characteristics and their own discipline, it necessarily raises the possibility that shared methods variance (i.e., correlated errors) may account for a portion of the correlation among the related variables. Future research could employ multi-informant and multi-method assessment, including observations of parenting and child characteristics to avoid these limitations. Third, our sample limited to Chinese normally developing preschool-aged children and their fathers and mothers. Future studies should examine to what extent the present results can be generalized to the younger or older children, clinically referred populations of young children, and families from different social and cultural backgrounds. Fourth, the low to moderate internal reliability of the scales used in the current study should be acknowledged. This may have attenuated the relations among study variables. Thus, it would be beneficial for future longitudinal studies to replicate the findings using more reliable measures. Finally, it should also be noted that the variables included in the present analysis accounted for only a small amount of variance in the measure of paternal and maternal harsh discipline. This suggests that other factors not measured in the present study may contribute to risks of Chinese parental harsh discipline, such as parental parenting stress and childhood history of discipline (Wang et al., 2014; Liu and Wang, 2015). In addition, the role of genetic and biological factors in determining levels of parental harsh discipline toward children also needs to be considered in future research (DiLalla and Gottesman, 1991).

Despite these limitations, some valuable information and important practical implications can be derived from our findings. First, this study presents new evidence suggesting links between children's characteristics and parental harsh discipline in Chinese societies. The inclusion of fathers' and mothers' harsh discipline extends the literature in important ways. The findings also provide an important supplement and extension to previous examinations of the factors associated with Chinese parental use of harsh discipline and its mechanisms. Second, the present study found that children's negative emotionality would cause excessive distress and anxiety in parents, consequently leading to the occurrence of harsh discipline, suggesting both children's and parents' negative emotionality as the common cause of harsh discipline. Treatment programs aimed at changing harsh discipline practices of Chinese parents are likely to be more effective if intervention efforts focus not only on parents' but also on children's emotion management. In addition, enhancing parents' knowledge and understanding of their child's temperament, as well as promoting parents' coping skills on how to handle children's negative emotionality in adaptive way may also be effective in decreasing parental harsh discipline.

## Ethics statement

This study was carried out in accordance with the recommendations of the Ethics Committee of Educational College of Capital Normal University with written informed consent from all subjects. All subjects gave written informed consent in accordance with the Declaration of Helsinki. The protocol was approved by the Ethics Committee of Educational College of Capital Normal University.

## Author contributions

All authors listed, have made substantial, direct and intellectual contribution to the work, and approved it for publication.

### Conflict of interest statement

The authors declare that the research was conducted in the absence of any commercial or financial relationships that could be construed as a potential conflict of interest.
